# COVID-19 Vaccination and Parent-Reported Symptomatic Child Asthma Prevalence

**DOI:** 10.1001/jamanetworkopen.2024.19979

**Published:** 2024-07-03

**Authors:** Matthew M. Davis, Lakshmi K. Halasyamani

**Affiliations:** 1 Nemours Children’s Health, Wilmington, Delaware; 2 Endeavor Health, Evanston, Illinois; 3 University of Chicago Pritzker School of Medicine, Chicago, Illinois

## Abstract

This cross-sectional study evaluates the association between COVID-19 vaccination and symptomatic child asthma.

## Introduction

Asthma was considered a risk for COVID-19 infection and hospitalization early in the pandemic.^1^ Social distancing measures in 2020 were associated with lower rates of emergency visits and hospitalizations for asthma among children.^2^ Individual-level risk of COVID-19 infection was reduced with vaccination against SARS-CoV-2 for adults and children in 2020 and 2021, and several states sustained other infection prevention efforts (eg, face mask requirements) into 2021.^3^

Whether symptomatic asthma among children was associated with population-level COVID-19 illness exposure or mitigation strategies is not understood. We hypothesized that symptomatic asthma would be positively associated with population-level COVID-19 overall mortality (a proxy for SARS-CoV-2 exposure), and would be inversely associated with population-level completion of the COVID-19 primary vaccination series and with state face mask mandates.

## Methods

This cross-sectional study followed the Strengthening the Reporting of Observational Studies in Epidemiology (STROBE) reporting guideline. The institutional review board of Nemours Children’s Health determined that the study was exempt from human participants review and the need for informed consent because data were publicly available. We used state-level data regarding parent-reported current asthma symptom prevalence in their children (National Survey of Children’s Health, 2018-2019 and 2020-2021); age-adjusted COVID-19 mortality rates (US Centers for Disease Control and Prevention [CDC]; 2020 and 2021); proportion of population aged 5 years and older who completed the COVID-19 primary vaccination series in 2020 to 2021 (CDC); and face mask requirements in enclosed spaces through August 2021 (20 states and District of Columbia).^3^

We calculated state-level change scores for parent-reported childhood asthma symptom prevalence for 2020 to 2021 vs 2018 to 2019 and then assessed state-level time trends using *t* tests. We evaluated trend associations with concurrent state-level variables (pairwise Pearson correlations and linear regression). All analyses were performed with StataSE version 16 (StataCorp), with statistical significance defined as 2-tailed α = .05. Data were analyzed in February 2024.

## Results

Mean state-level prevalence of parent-reported childhood asthma symptoms decreased from 7.77% (95% CI, 7.34%-8.21%) in 2018 to 2019 to 6.93% (95% CI, 6.53%-7.32%) in 2020 to 2021 (*P* < .001). The absolute mean (SD) change score was −0.85 (1.26) percentage points (Table).

**Table.** State-Level Prevalence of Parent-Reported Current Asthma Symptoms in Children, in 2018 to 2019 and 2020 to 2021^a^

State	Current asthma symptom prevalence, %		Prevalence change, 2020-2021 vs 2018-2019, percentage points
	2018 to 2019	2020 to 2021	
Alabama	9.1	8.5	−0.6
Alaska	6.1	5.5	−0.6
Arizona	6.9	6.6	−0.3
Arkansas	7.9	8.5	0.6
California	7.4	5.3	−2.1
Colorado	8.0	6.9	−1.1
Connecticut	10.0	9.6	−0.4
Delaware	10.2	9.6	−0.6
District of Columbia	11.6	8.1	−3.5
Florida	7.7	6.9	−0.8
Georgia	8.5	9.6	1.1
Hawaii	7.1	6.2	−0.9
Idaho	6.1	4.9	−1.2
Illinois	7.8	6.3	−1.5
Indiana	6.8	6.7	−0.1
Iowa	6.4	5.6	−0.8
Kansas	8.5	7.8	−0.7
Kentucky	5.8	6.3	0.5
Lousiana	8.6	6.6	−2.0
Maine	9.8	6.7	−3.1
Maryland	10.1	7.9	−2.2
Maine	8.5	7.8	−0.7
Michigan	9.1	7.8	−1.3
Minnesota	7.0	5.2	−1.8
Mississippi	10.6	10.2	−0.4
Missouri	8.3	6.6	−1.7
Montana	8.5	6.3	−2.2
Nebraska	7.2	4.3	−2.9
Nevada	7.3	5.4	−1.9
New Hampshire	7.5	6.4	−1.1
New Jersey	9.3	6.1	−3.2
New Mexico	6.6	9.3	2.7
New York	9.4	8.0	−1.4
North Carolina	7.1	5.7	−1.4
North Dakota	5.0	5.6	0.6
Ohio	7.9	7.1	−0.8
Oklahoma	9.6	9.0	−0.6
Oregon	5.9	6.2	0.3
Pennsylvania	8.4	9.0	0.6
Rhode Island	9.9	7.4	−2.5
South Carolina	8.5	7.9	−0.6
South Dakota	5.9	5.3	−0.6
Tennessee	7.6	5.6	−2.0
Texas	6.5	7.1	0.6
Utah	4.3	5.9	1.6
Vermont	7.1	7.0	−0.1
Virginia	7.3	5.4	−1.9
Washington	7.1	6.1	−1.0
West Virginia	6.7	6.1	−0.6
Wisconsin	5.2	6.6	1.4
Wyoming	6.7	6.7	0.0
US	7.77	6.93	−0.85

^a^ Parents’ affirmative answers regarding current asthma symptom prevalence were based on yes answers to a 2-question sequence in the National Survey of Children’s Health regarding 1 randomly selected child in the household: (1) “Has a doctor or health care provider EVER told you that this child has asthma?”; and (2) If yes, “Does this child CURRENTLY have the condition?” Two-year combinations were used to generate robust prevalence estimates within each state, as recommended by the Data Resource Center for Child and Adolescent Health.

The mean (SD) age-adjusted state-level COVID-19 mortality rate was 80.3 (30.2) per 100 000 population in 2020, increasing to 99.3 (33.9) in 2021. The mean (SD) state-level COVID-19 primary series vaccination rate through December 2021 was 72.3% (10.3%).

Based on linear regression, with each increase of 10 percentage points in COVID-19 vaccination coverage, parent-reported child asthma symptom prevalence decreased by 0.36 percentage points (*P* = .04) (Figure). Parent-reported child asthma symptom prevalence was not associated with state-level COVID-19 mortality or with face mask requirements. State-level COVID-19 vaccination rates were inversely correlated with the state-level COVID-19 mortality rate in 2021 (*r* = −0.75; *P* < .001) but not in 2020 (*r* = −0.20; *P* = .16), and were positively correlated with face mask mandates (*r* = .49; *P* < .001).

**Figure.**
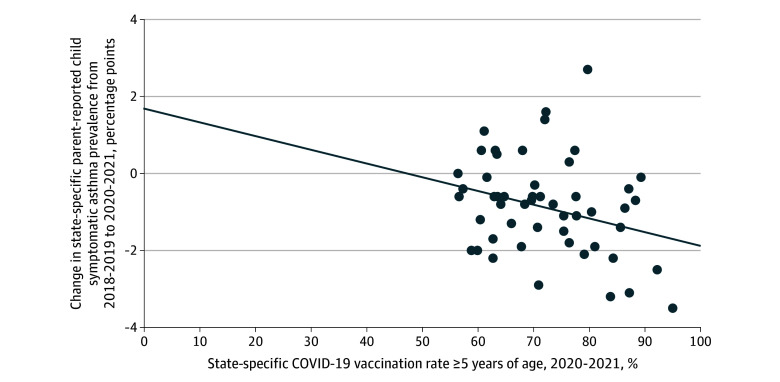
State-Level Change in Prevalence of Parent-Reported Current Asthma Symptoms in Their Children From 2018-2019 to 2020-2021, vs Corresponding State-Level COVID-19 Vaccination Rate for Persons Aged 5 Years or Older Reflecting the Primary Series Received in 2020 to 2021 Each dot represents a state or the District of Columbia. The regression line for the inverse association between state-level change in parent-reported current symptomatic asthma prevalence among their children and state-level COVID-19 vaccination rate is shown (*P* = .04).

## Discussion

In this study, which is the first population-level parent-reported childhood asthma symptom prevalence and COVID-19 vaccination study we know of, we found that higher COVID-19 vaccination rates may confer protection against symptomatic asthma. COVID-19 vaccination yields prophylactic benefits against SARS-CoV-2 infection for individual children^4^ and may also protect against other human coronaviruses through cross-reactive antibody responses.^5^ Community-level immunity in states with higher vaccination rates may have helped reduce children’s asthma risk. In contrast, neither concurrent exposure to high population-level burden of COVID-19–attributed disease nor sustained state-level face mask requirements were associated with concurrent trends in parent-reported symptomatic childhood asthma.

A key limitation of this analysis is that state-level estimates of COVID-19 vaccination rates among children with a history of asthma are not available. Therefore, we could not assess for differences in symptomatic asthma among vaccinated vs unvaccinated children. Nonetheless, reduction in symptomatic asthma among children in 2020^1^ and overall individual-level COVID-19 mortality reduction with vaccination against SARS-CoV-2^6^ offer external support for our state-level findings. Moreover, the absence of association of COVID-19 vaccination (administered predominantly in 2021) with population-level COVID-19 mortality in 2020 serves as a negative control. These findings merit further assessment to determine whether childhood asthma symptom prevalence may be reduced by sustained vaccination efforts against SARS-CoV-2.

## Notes

Supplement.
Data Sharing Statement
