# Supplementary material for: COVID-19 Vaccination and Parent-Reported Symptomatic Child Asthma Prevalence
Source: JAMA Netw Open. 2024 Jul 3;7(7):e2419979. doi: 10.1001/jamanetworkopen.2024.19979 (PMC11222989; doi:10.1001/jamanetworkopen.2024.19979)
Supplement: Supplement. — Data Sharing Statement [file jamanetwopen-e2419979-s001.pdf]

Data Sharing Statement

Davis. Association of COVID-19 Vaccination With Parent-Reported Symptomatic Child Asthma Prevalence. *JAMA Netw Open*. Published July 03, 2024.
doi:10.1001/jamanetworkopen.2024.19979

Data
Data available: Yes
Data types: Data (not involving human participants)
How to access data: matthew.davis@nemours.org
When available: With publication

Supporting Documents
Document types: None

Additional Information
Who can access the data: researchers
Types of analyses: for a specified purpose
Mechanisms of data availability: after approval of a proposal
Any additional restrictions: none
